# County-level intensity of carbon emissions from crop farming in China during 2000–2019

**DOI:** 10.1038/s41597-024-03296-y

**Published:** 2024-05-06

**Authors:** Cheng Li, Junwen Jia, Fang Wu, Lijun Zuo, Xuefeng Cui

**Affiliations:** 1 Department of Ecology, School of Plant Protection / Joint International Research Laboratory of Agriculture and Agri-Product Safety of the Ministry of Education, Yangzhou University, Yangzhou, 225009 China; 2 School of Systems Science, Beijing Normal University, Beijing, 100875 China; 3 School of Earth and Environmental Sciences, Cardiff University, Cardiff, CF10 3AT United Kingdom; 4 Aerospace Information Research Institute, Chinese Academy of Sciences, Beijing, 100093 China

**Keywords:** Climate sciences, Ecology

## Abstract

Agriculture is an important contributor to global carbon emissions. With the implementation of the Sustainable Development Goals of the United Nations and China’s carbon neutral strategy, accurate estimation of carbon emissions from crop farming is essential to reduce agricultural carbon emissions and promote sustainable food production systems in China. However, previous long-term time series estimates in China have mainly focused on the national and provincial levels, which are insufficient to characterize regional heterogeneity. Here, we selected the county-level administrative district as the basic geographical unit and then generated a county-level dataset on the intensity of carbon emissions from crop farming in China during 2000–2019, using random forest regression with multi-source data. This dataset can be used to delineate spatio-temporal changes in carbon emissions from crop farming in China, providing an important basis for decision makers and researchers to design agricultural carbon reduction strategies in China.

## Background & Summary

The rapid increase in greenhouse gas emissions such as CO_2_, CH_4_, and N_2_O has further exacerbated the global warming process, seriously threatening the sustainable development of human society^1–3^. As a basic industry of the national economy, agriculture is easily affected by global warming, which can have a significant impact on crop farming and contribute further to the instability of crop production and supply^4,5^. Meanwhile, crop farming is also one of the major sources of global greenhouse gas emissions of CO_2_ and non-CO_2_, and therefore acts as a crucial role in global warming^6–8^. China is the world’s leading agricultural producer^9^, which feeds 20% of the global population with only 7% of global croplands by securing arable land and increasing fertilizer and pesticide inputs over the past decades^10^. However, the fact that these main measures can further increase carbon emissions has been a great concern^11–13^. With the implementation of the Sustainable Development Goals of the United Nations and China’s carbon neutral strategy^14,15^, a detailed understanding of the temporal and spatial changes in China’s carbon emissions from crop farming is essential to reduce agricultural carbon emissions and promote sustainable food production systems in China.

There are several carbon emissions datasets related to crop farming at different spatial levels around the world. The Food and Agriculture Organization of the United Nations (FAO) provided a complete and coherent time series of emission statistics at national scale since 1961^16^. Furthermore, Carlson *et al*.^17^ developed the 5-arc-minute crop-specific circa 2000 estimates of carbon emissions, reporting the effects of rice paddy management, peatland draining, and nitrogen fertilizer on CH_4_, CO_2_ and N_2_O. In China, Zuo *et al*.^18^ calculated carbon emissions from agricultural management (e.g., fertilizer application, rice cultivation, peatland draining) and cropland changes for 1987, 2000, and 2010. Zhang *et al*.^19^ calculated provincial-level carbon emissions of grain production from 1997 to 2020, including emissions from material inputs, straw burning, fertilizer application, and rice cultivation. In comparison, Liang *et al*.^20^ reconsidered primary and secondary emissions from various agricultural activities involved in crop farming, in which crop residue open burning, rice cultivation, cropland change, cropland emissions, machinery use, nitrogen fertilizer production, and pesticide production were included, and then calculated provincial-level carbon emissions from 1978 to 2016 in China. These existed studies have mainly focused on carbon emissions at the national and provincial levels, and have taken into account different agricultural activities, parameters and study periods in their calculations, making comparisons difficult in China^18–21^. A recent study indicated that the spatial heterogeneity of agricultural emissions highlighted necessity of tailored regional mitigation strategies in China^22^. Although counties are the most basic government units in China and their important role in the implementation of national policies^23^, few studies have been conducted at the county level due to the low availability of data sources in China.

To fill those knowledge gaps, this study contributes the following work to this research area: (1) we updated provincial-level carbon emissions from crop farming during 1978–2019 in China based on Liang *et al*.^20^, and calculated the corresponding intensity of carbon emissions; and (2) we used the random forest-based downscaling method to calculate the county-level intensity of carbon emissions from crop farming during 2000–2019 in China. The results of this study can help to delineate the spatio-temporal changes in carbon emissions from crop farming in China and provide an important basis to design China’s agricultural carbon reduction strategies.

## Methods

### Calculation of the intensity of provincial-level carbon emissions from crop farming

Although Liang *et al*.^20^ provided a comprehensive and long-term dataset on provincial-level carbon emissions from crop farming in China during 1978–2016 (Figure S1), there is a great uncertainty in the activity level data for cropland change^24,25^, derived from satellite land use/cover data for 1980, 1990, 2000, 2010, and 2015 in their work^26^ (Figure S1c). Moreover, there are also large differences in cropland changes compared to those from other commonly used land use/cover datasets (Figure S2). In order to reduce uncertainty, we consider emissions from the following six agricultural activities except for emissions from cropland change, including crop residue open burning, rice cultivation, cropland emissions, machinery use, nitrogen fertilizer production, and pesticide production.

**Fig. 1:**
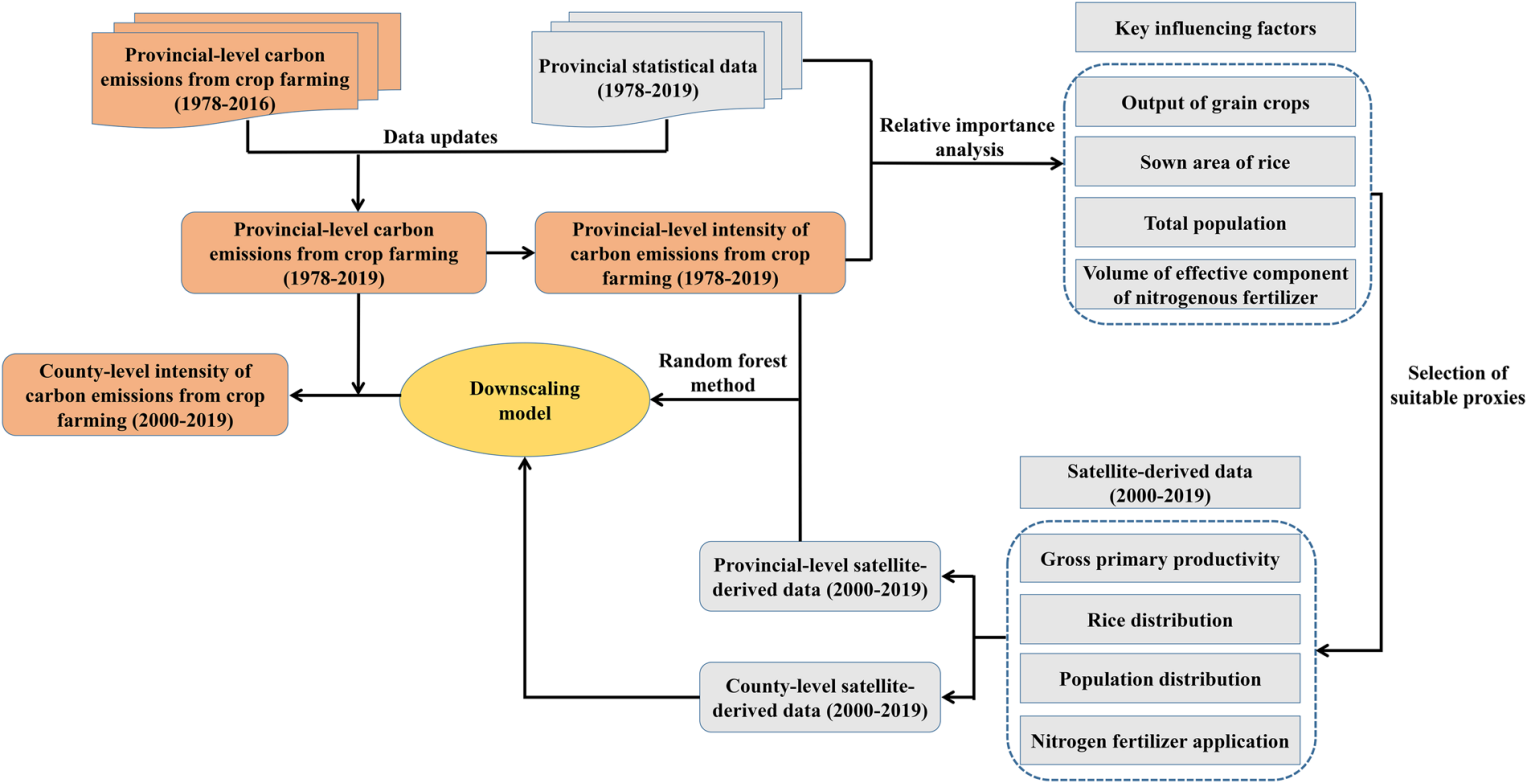
Workflow of the study.

According to data sources, emission factors, and calculation methods provided by Liang *et al*.^20^, we first updated provincial-level carbon emissions from crop farming during 1978–2019 in China. We then further calculated the intensity of provincial-level carbon emissions from crop farming (Unit: ton CO_2_-eq/km^2^) based on the land area of each province (Fig. 1).

### Selection of important indicators affecting the intensity of carbon emissions from crop farming

Based on provincial statistics from the National Bureau of Statistics of China, we selected 12 indicators that could affect carbon emissions from crop farming (Table 1). Furthermore, the results per unit area were calculated for these indicators based on the land area of each province, which were set as independent variables. In this study, the intensity of provincial-level carbon emissions from crop farming was taken as the dependent variable, a random forest method was used to identify key factors that influence the intensity of provincial-level carbon emissions from crop farming^27–29^ (Fig. 1). As shown in Table 1, the output of grain crops, sown area of rice, total population, and volume of effective component of nitrogenous fertilizer had significant impacts on the intensity of provincial-level carbon emissions from crop farming, and their overall magnitudes of relative importance reached 95.76%. For these important influencing factors (Table 1), we selected suitable proxies based on available satellite-derived datasets in this work^30–33^.

**Table 1:** An overview of 12 indicators that could affect the intensity of provincial-level carbon emissions from crop farming.

Indicators	Relative importance (%)	Suitable proxy indicators
Output of grain crops	2.40	Gross primary productivity^30^
Sown area of rice	36.44	Rice distribution^31^
Total population	50.32	Population distribution^32^
Volume of effective component of nitrogenous fertilizer	6.60	Nitrogen fertilizer application^33^
Use of agricultural pesticide	1.13	/
Number of large animals	1.20	/
Number of sheep and goats	0.25	/
Total power of agricultural machinery	0.20	/
Number of large and medium-sized agricultural tractors	0.32	/
Number of small tractors	0.39	/
Gross output value of agriculture, forestry, animal husbandry and fishery	0.27	/
Gross output value of agriculture	0.48	/

### Calculation of the intensity of county-level carbon emissions from crop farming

Based on the intensity of provincial-level carbon emissions from crop farming and satellite-derived data in China from 2000 to 2019, we constructed a downscaling model using the random forest method^34,35^ (Fig. 1). In order to evaluate the validation, we randomly divided 10% of the sample data into a testing set using for Leave-One-Out validation, and the remaining sample data was used as a training set^36,37^. Among the training set, we chose 10% of the data randomly at one time for 10-fold cross-validation to avoid over-fitting^38,39^. Two evaluation indicators were used to evaluate the performance of the simulation, including the determination coefficients (*R*^2^) and the root mean square error (RMSE). As shown in Table 2, our constructed downscaling model provided the superior performance in both the training and testing sets, indicating that this model could reflect the intensity of carbon emissions from crop farming in China.

**Table 2:** Evaluation indicators for the downscaling model on the intensity of provincial-level carbon emissions from crop farming.

Datasets	Evaluation indicators	Downscaling model
Training set	*R*^2^	0.98
	RMSE	5.6 × 10^−4^
Testing set	*R*^2^	0.99
	RMSE	4.2 × 10^−4^

We then put the satellite-derived county-level data in China from 2000 to 2019 into the downscaling model, and used provincial-level carbon emissions of crop farming as a constraint to adjust the county-level carbon emissions for each province^40^. At last, we generated a county-level dataset on the intensity of carbon emissions from crop farming in China during 2000–2019 (Fig. 1).

## Data Records

**Fig. 2:**
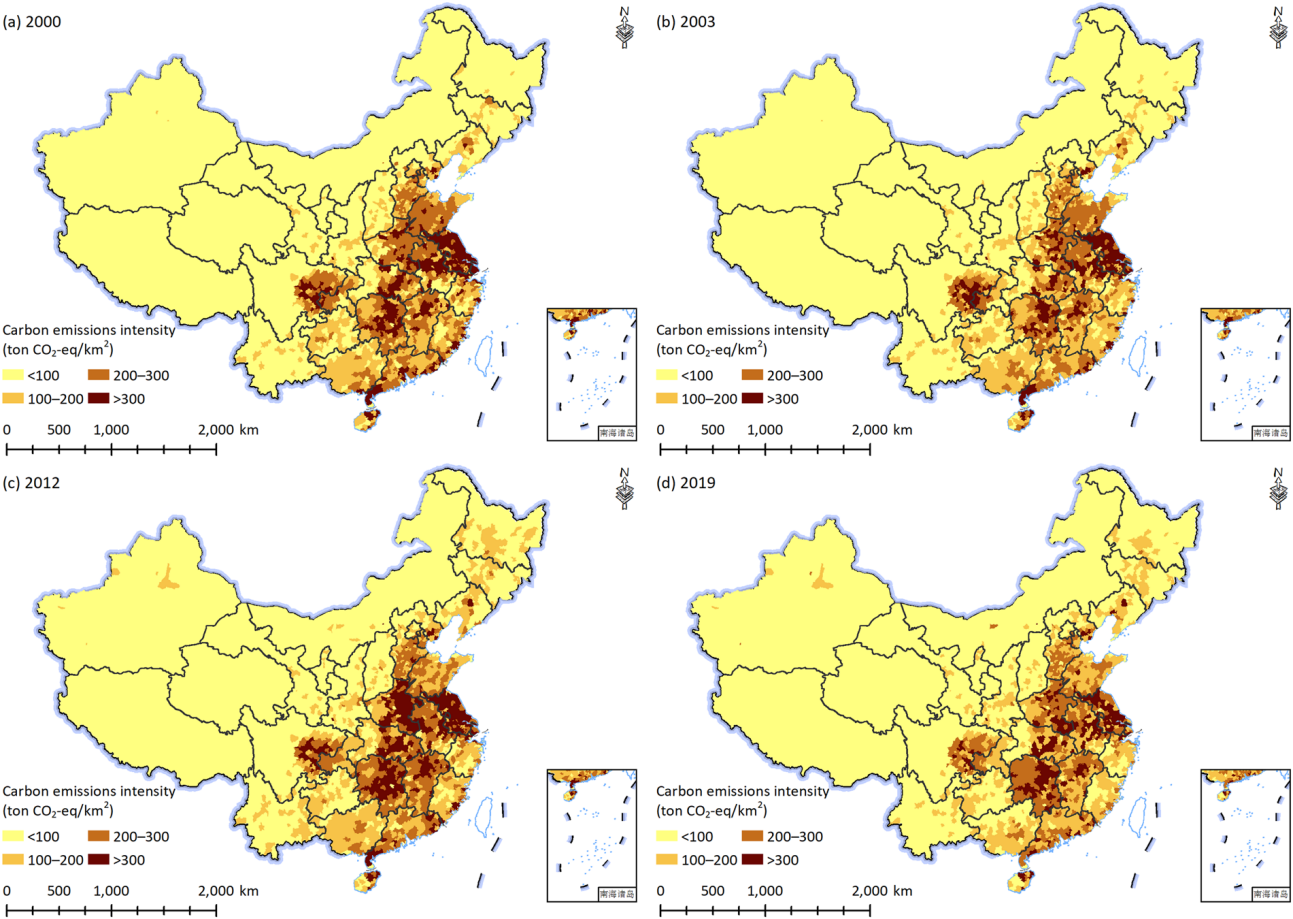
Spatial patterns of county-level intensity of carbon emissions from crop farming in China during 2000–2019.

The dataset available on the Figshare^41^ consists of two components. One is an Excel file describing the intensity of county-level carbon emissions from crop farming in China during 2000–2019. The other is the vector map, which can be used in the ArcGIS software to display all results by matching the Excel file. The temporal and spatial changes in the intensity of county-level carbon emissions from crop farming in China are shown in Fig. 2.

## Technical Validation

### Validity testing for time series of carbon emissions from crop farming in China

**Fig. 3:**
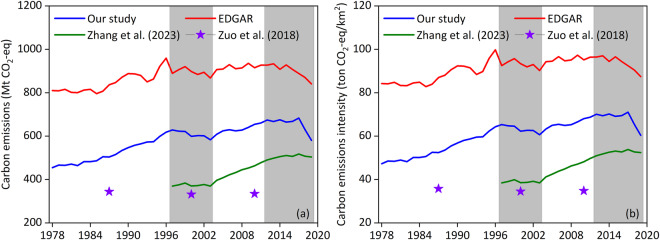
Changes in carbon emissions from crop farming (**a**) and its intensity (**b**) in China.

Based on the calculation method described above, we obtained provincial-level carbon emissions from crop farming during 1978–2019, and further calculated the corresponding intensity of carbon emissions from crop farming according to the land area of each province (Fig. 2). In order to compare our results with other studies, the Emissions Database for Global Atmospheric Research (EDGAR) dataset^42^ and previous accounting results^18–20^ were also collected and shown in Fig. 3.

At the national level, the results of our accounting showed an overall upward trend in carbon emissions from crop farming in China during 1978–2019, but there was a significant decline between 1997 and 2003 and between 2012 and 2020, respectively (Fig. 3a). Similar characteristics were also shown in the intensity of carbon emissions from crop farming in China (Fig. 3b), which were consistent with the EDGAR dataset^42^ and previous accounting results^18–20^. However, direct numerical comparisons were difficult, considering that these studies selected different agricultural activities^20^.

### Validity testing for the intensity of county-level carbon emissions from crop farming in China

**Fig. 4:**
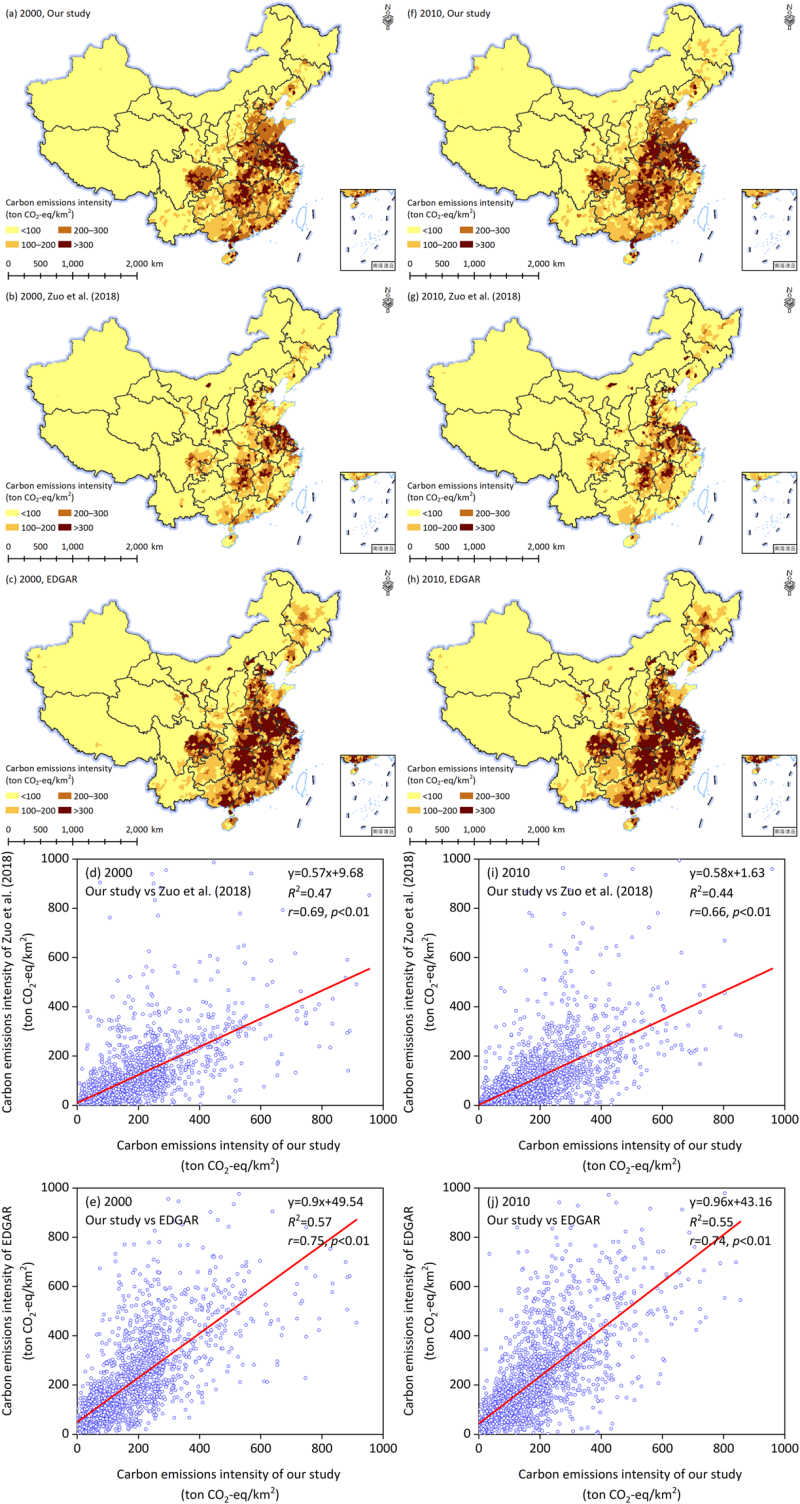
Comparison of carbon emissions intensity estimated from Zuo *et al*.^18^ and the EDGAR dataset for 2000 (**a**–**e**) and 2010 (**f**–**j**).

At the spatial scale, we compared our findings with those of Zuo *et al*.^18^ and the EDAGR dataset for 2000 and 2010 in China (Fig. 4), which are raster data with a spatial resolution of 10 km. For comparison purposes, we extracted the results of Zuo *et al*.^18^ and the EDAGR dataset for each county in China using ArcGIS 10.2 software.

As shown in Fig. 4a and f, the results of our accounting showed an obvious spatial heterogeneity in the intensity of county-level carbon emissions from crop farming, with an overall distribution pattern of higher in the south and east and lower in the west and north. These spatial patterns were consistent with those of Zuo *et al*.^18^ (Fig. 4b and g) and the EDAGR dataset (Fig. 4c and h). Furthermore, there were significant positive relationships between our findings and the results of Zuo *et al*.^18^ for 2000 and 2010 (Fig. 4d and i). Moreover, significant positive relationships were also found between our findings and the results of the EDGAR dataset for 2000 and 2010 (Fig. 4e and j). The correlation coefficients between our findings and the results of Zuo *et al*.^18^ for 2000 and 2010 were 0.69 and 0.66, respectively, which were lower than those between our findings and the EDGAR dataset. Moreover, the corresponding *R*^2^ values between our findings and the results of Zuo *et al*.^18^ for 2000 and 2010 were lower than those between our findings and the EDGAR dataset. In addition to the spatial pattern, the results of our accounting were higher than those of Zuo *et al*.^18^ (Fig. 4b and g), but lower than those of the EDGAR dataset. An important reason was that different scholars considered carbon emissions from different agricultural activities and parameters in their work, which made direct comparisons difficult. For example, Zuo *et al*.^18^ considered carbon emissions from agricultural management (e.g., fertilizer application, rice cultivation, peatland draining) and cropland changes, whereas the EDGAR dataset mainly considered agricultural carbon emissions. As previously described, we mainly focused on primary and secondary emissions from crop farming^20^, and provided a long-term and spatially-precise profile of carbon emissions intensity from cropping farming in China.

### Limitations and future work

The limitations of our dataset are based on three main aspects. First, our accounting system do not cover all agricultural activities involved in crop farming. For example, the contribution of CO_2_ emissions resulting from land use transitions, such as the conversion of natural habitats for agricultural purposes or changes in agricultural land use, is not included in this dataset due to the high level of uncertainty in China’s land use/land cover data^24,43^ (Figure S2). Additionally emissions from activities like forest product harvesting, peat drainage, and peat burning should be included as well^17,18^, however, relevant long-term statistical data are difficult to obtain and are therefore not considered at the current stage. Second, there are many factors influencing the intensity of provincial-level carbon emissions from crop farming. However, it is difficult to find the corresponding satellite data or county-level statistical data for all these influencing factors when we constructed the downscaling model using the random forest method, which affects our calculating results. Third, this dataset is limited by different time spans between the provincial statistical data and satellite-derived data. Currently, this dataset is from 2000 to 2019 due to the availability of satellite-derived rice distribution data^31^. In the future, we will utilize multi-source data and data-model fusion method to further evaluate emissions from cropland change and other activities related to agriculture, and dynamically update our current dataset.

## Usage Notes

In this study, we provide a county-level dataset on the intensity of carbon emissions from crop farming in China during 2000–2019, which can bridge existing gaps in the available datasets^18–20^. On the one hand, this dataset can be used to analyse spatio-temporal changes in carbon emissions from crop farming and their driving factors in China from a county-scale perspective. On the other hand, this dataset also provides an important basis for decision makers and researchers to design agricultural carbon reduction strategies suitable for local contexts in China.

It is noted that the study area is mainland China (excluding Hong Kong, Macao and Taiwan). A total of 2369 data records are included in this dataset because we take the city-governed districts as a whole.

### Supplementary information

Supplementary information

## Data Availability

Python and ArcGIS are the software used to generate all the results. The code used for the downscaling calculations is available on the Figshare^41^.
