# Supplementary material for: County-level intensity of carbon emissions from crop farming in China during 2000–2019
Source: Sci Data. 2024 May 6;11:457. doi: 10.1038/s41597-024-03296-y (PMC11074320; doi:10.1038/s41597-024-03296-y)
Supplement: Supplementary file 1 — Supplementary information [file 41597_2024_3296_MOESM1_ESM.docx]

**Supplementary Information**

Table of Contents

[Figure S1. **China’s carbon emissions from cropping system during 1978-2016 based on Liang *et al.*^1^** 2](#_Toc164574511)

[Figure S2. **Areas of cropland change in China based on four commonly used land use/land cover datasets** 3](#_Toc164574512)

[**References** 3](#_Toc164574513)


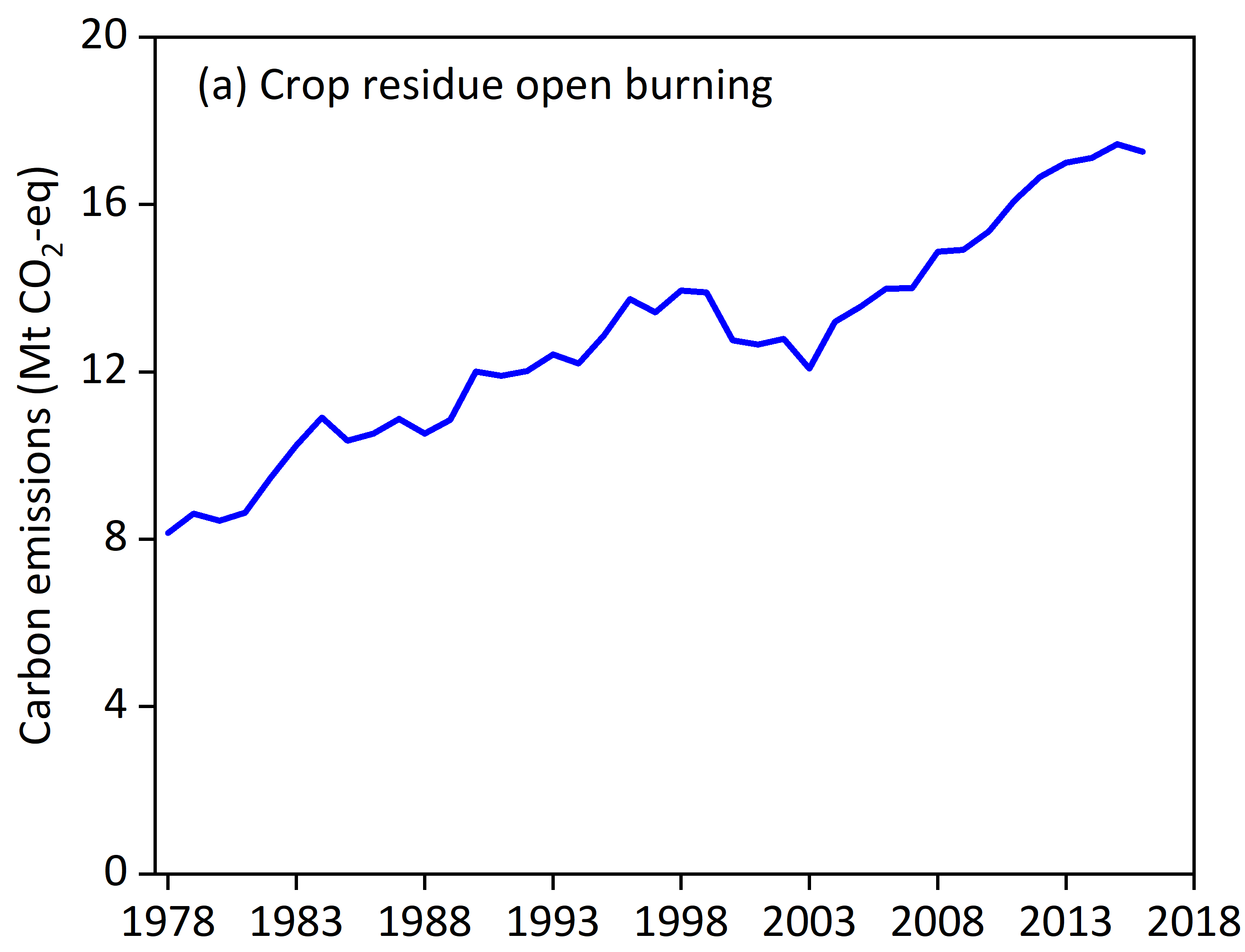

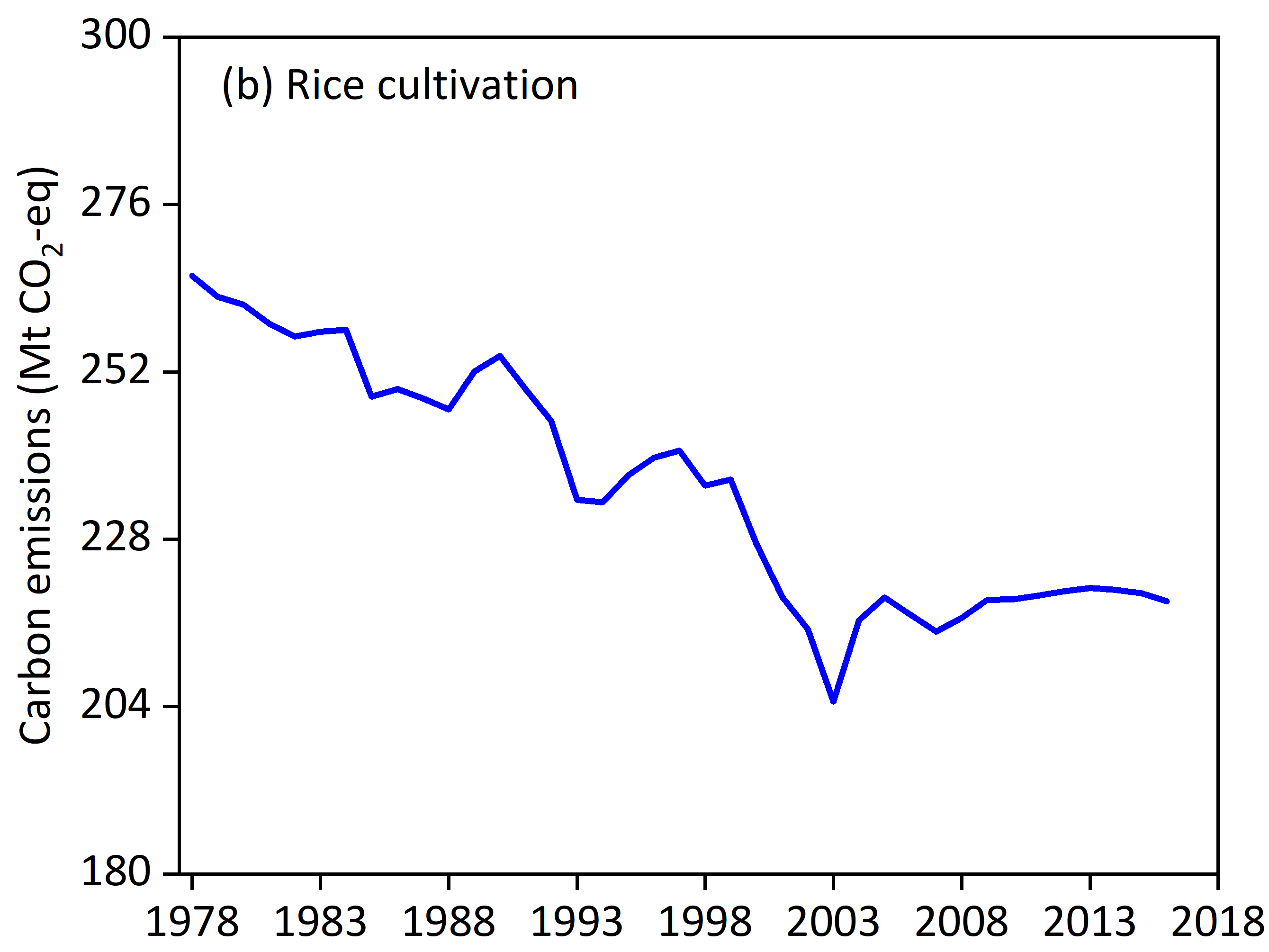

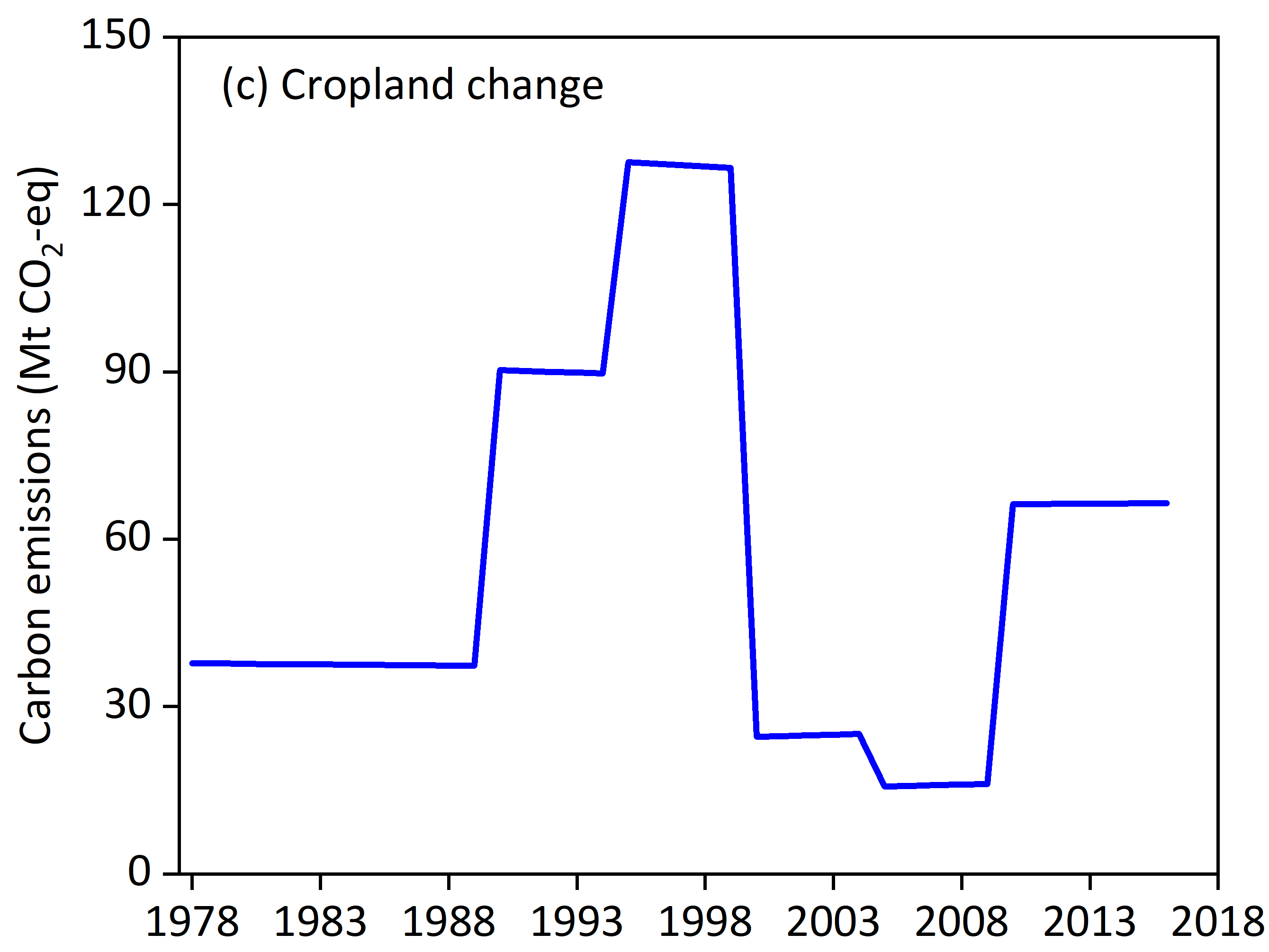

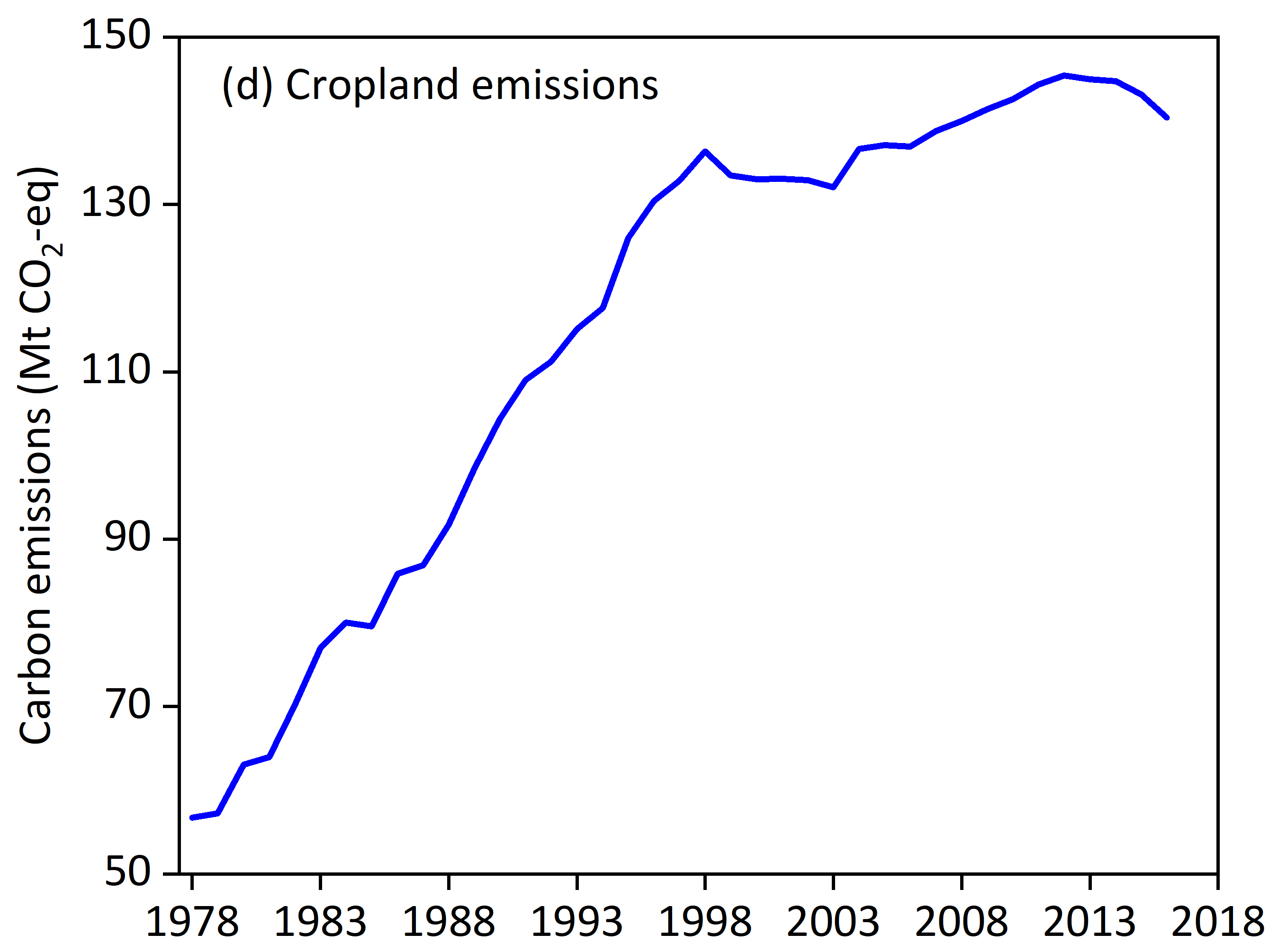

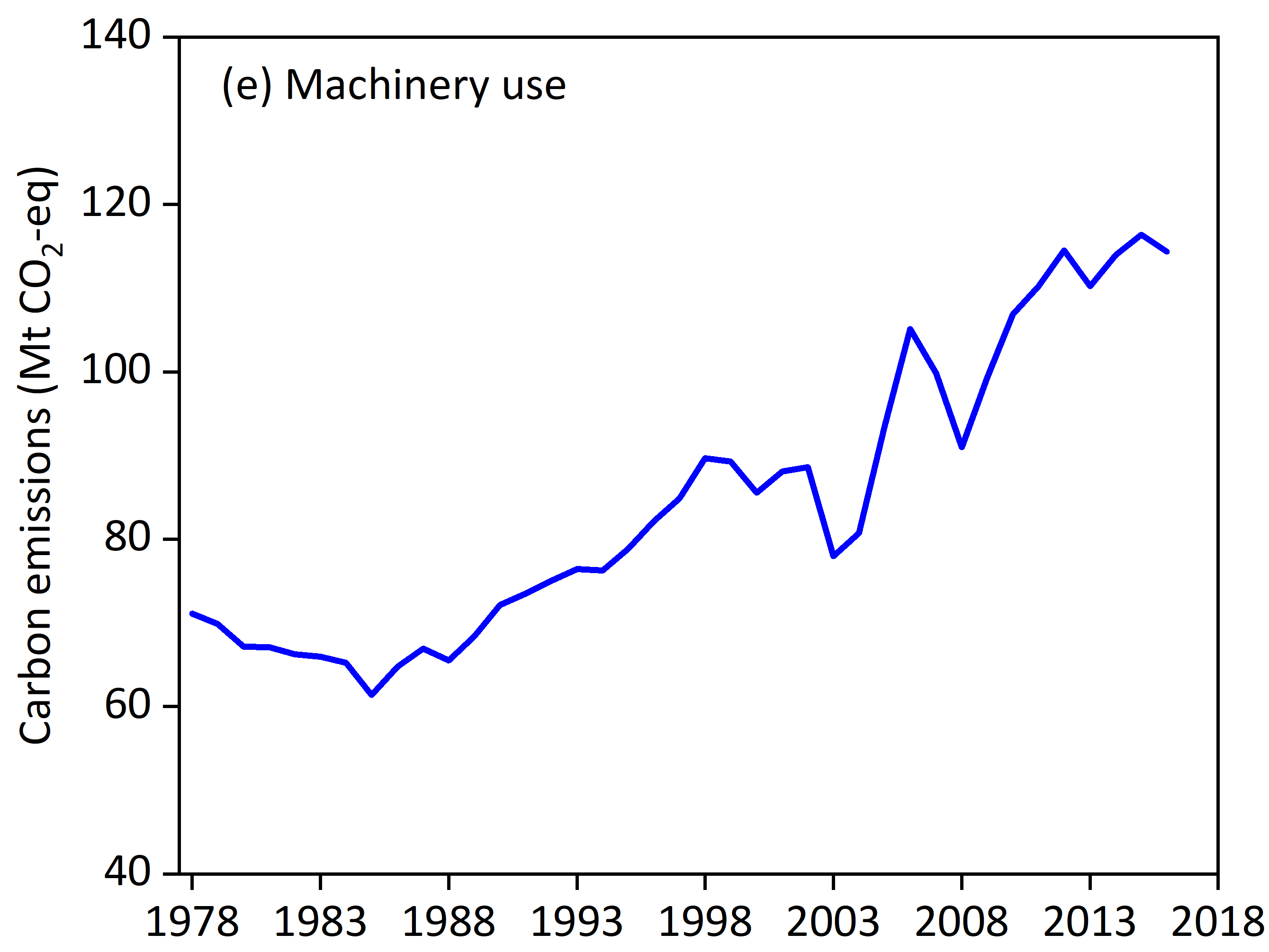

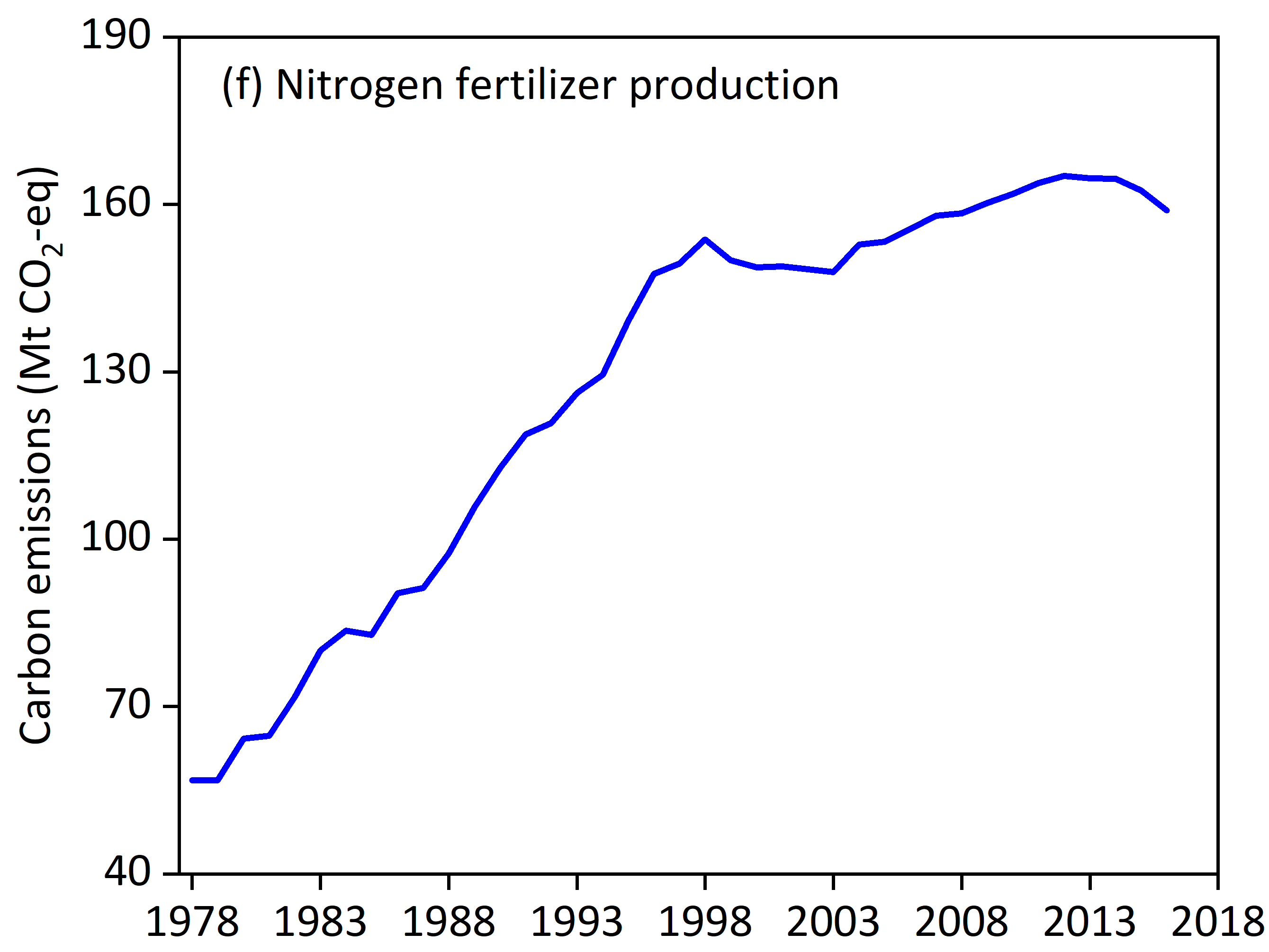

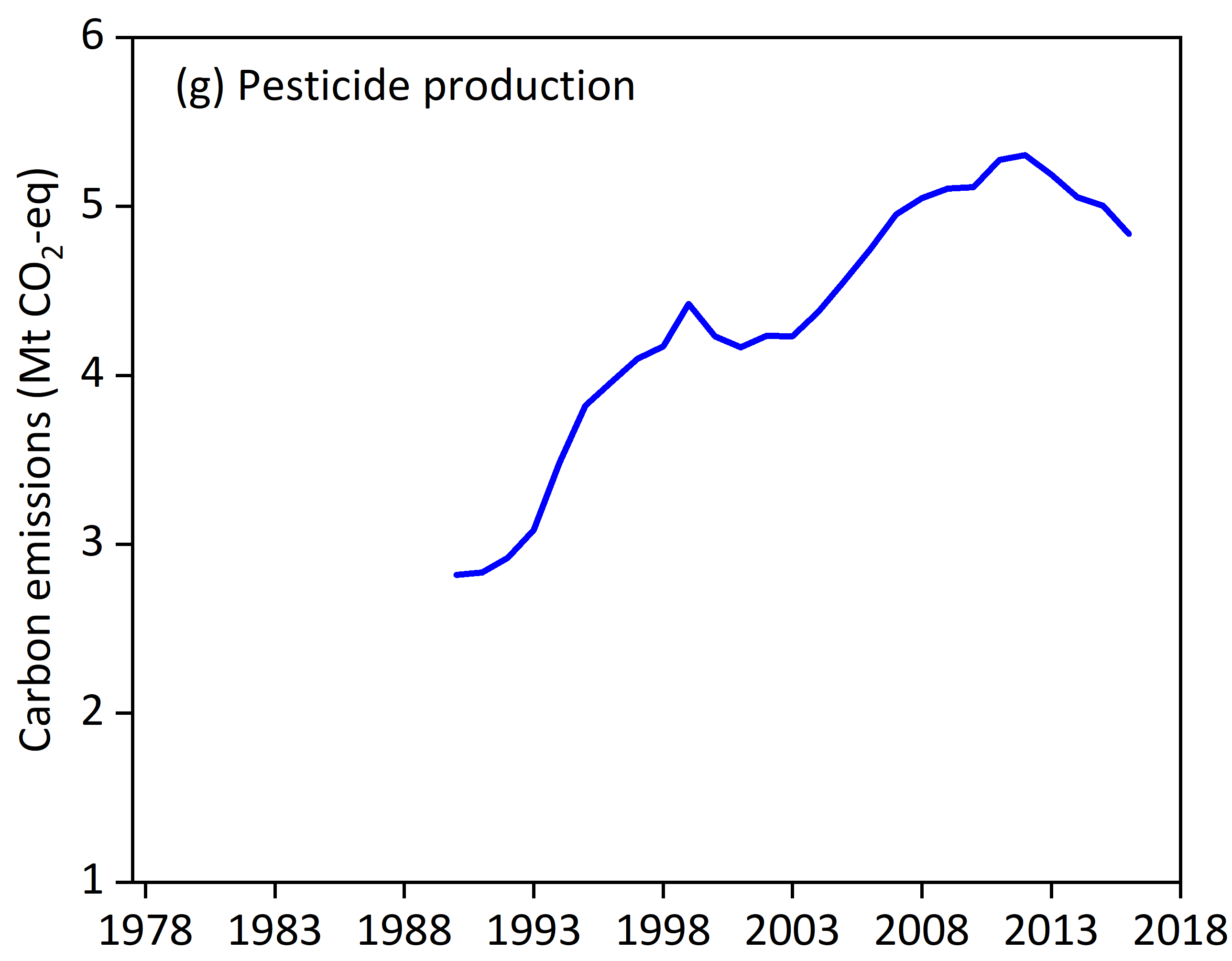


### Figure S1. **China’s carbon emissions from cropping system during 1978-2016 based on Liang *et al.*^1^**


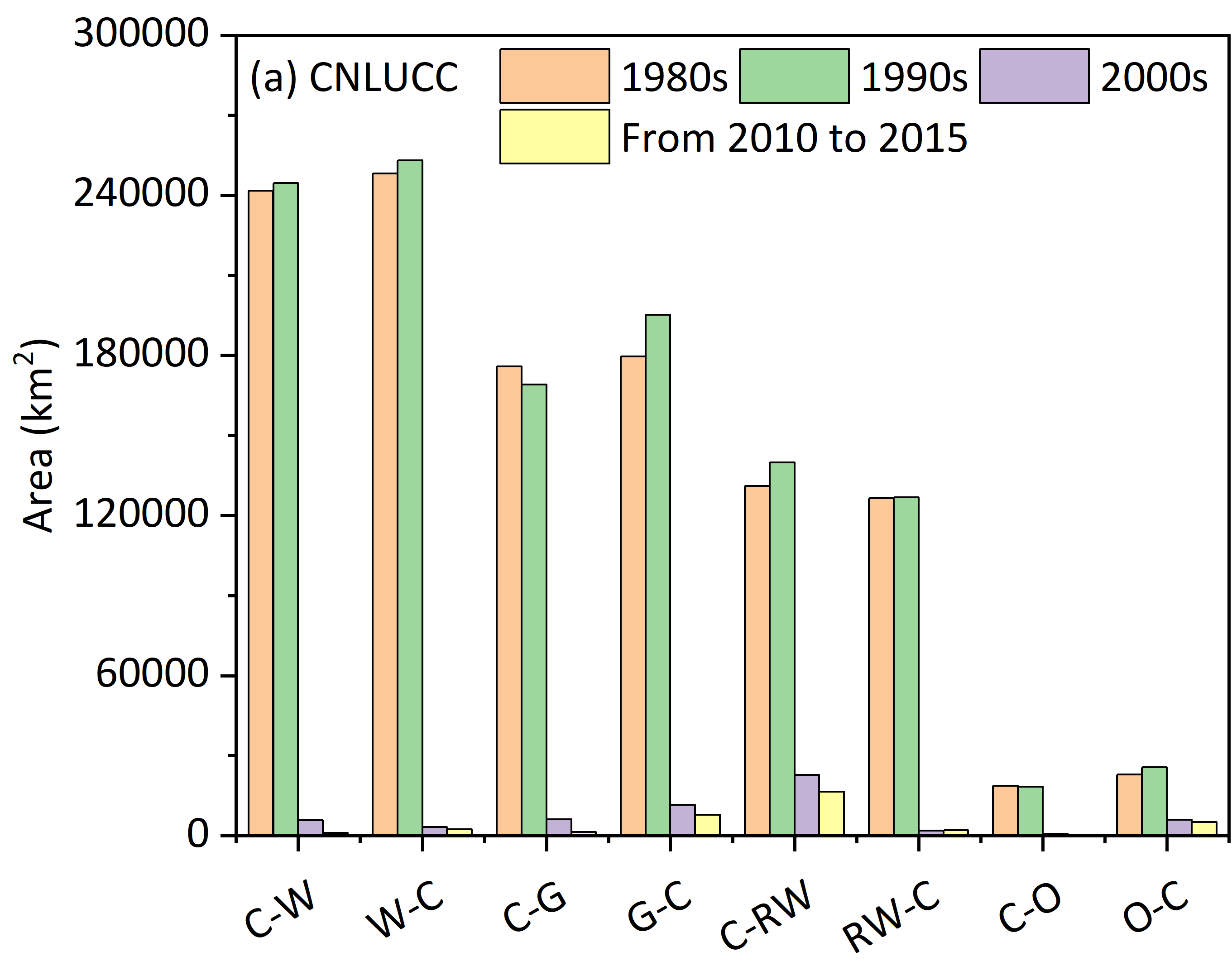

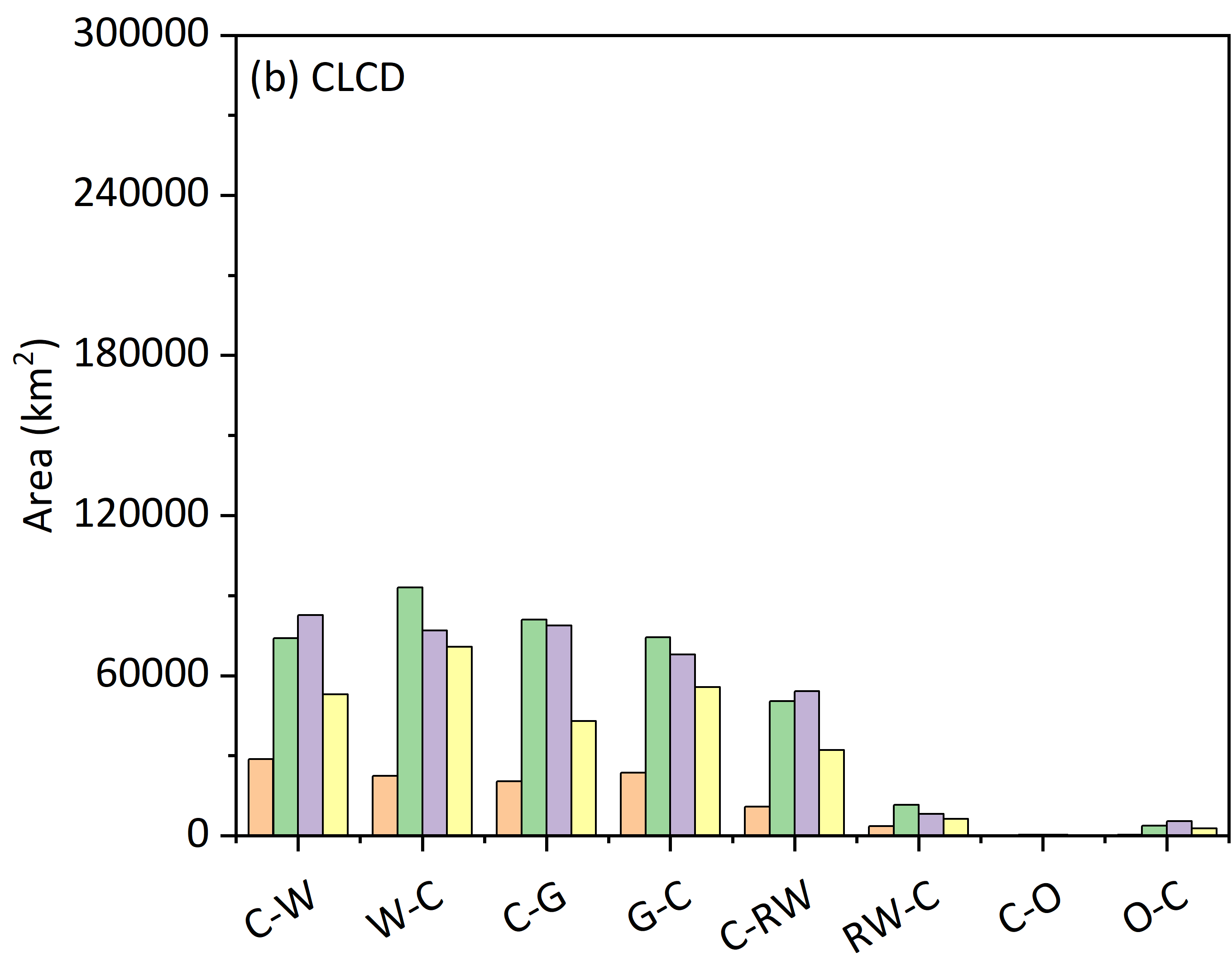

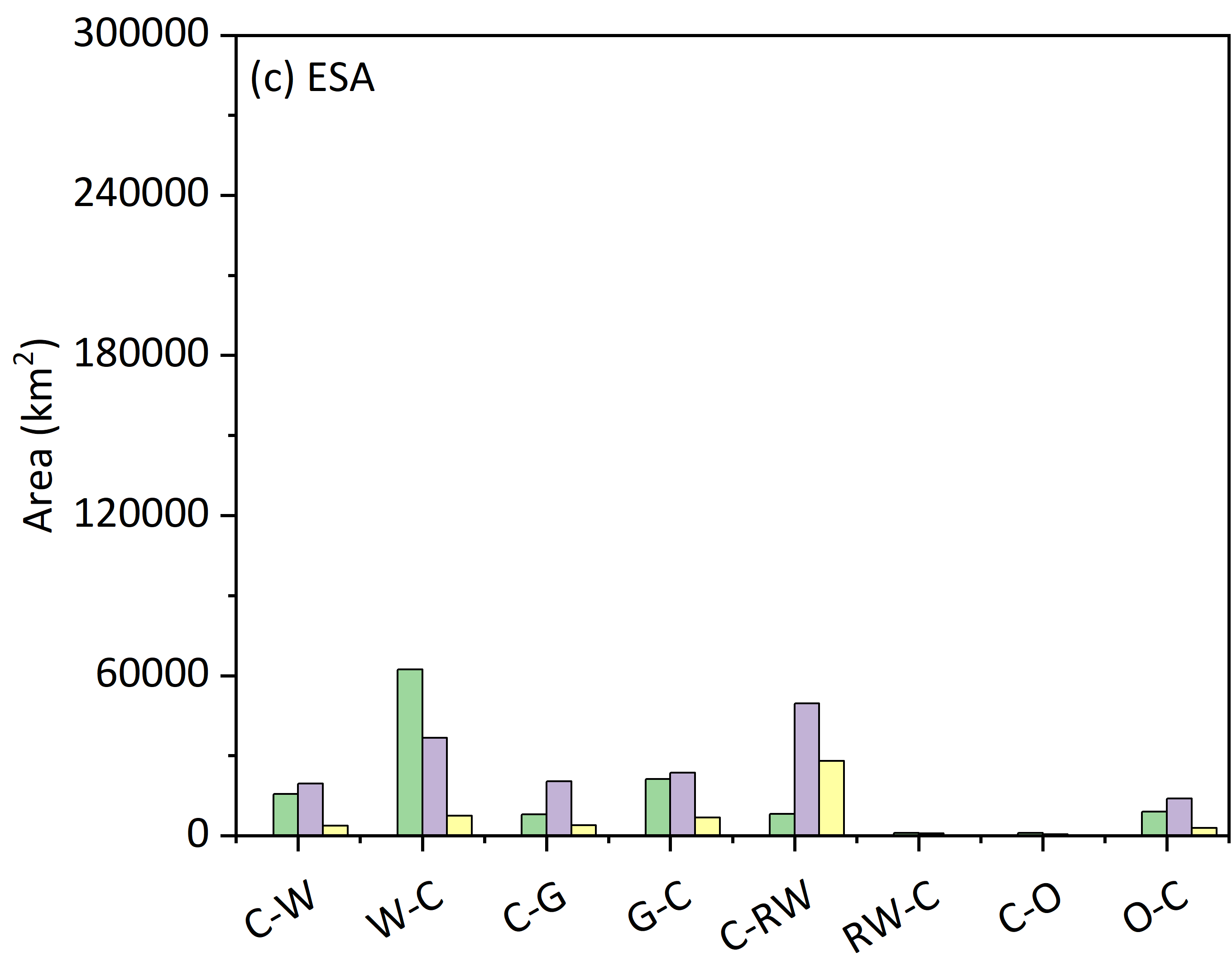

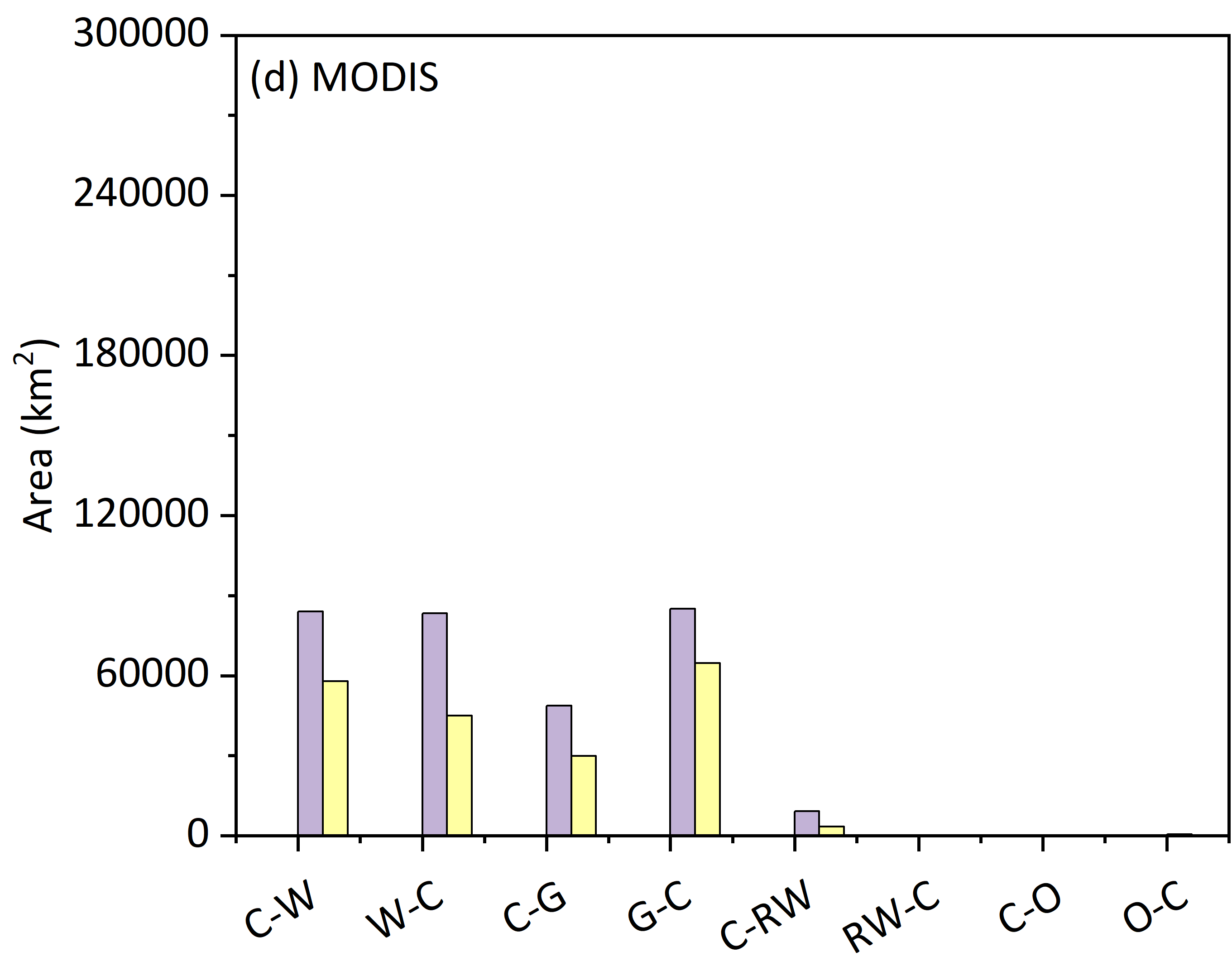


### Figure S2. **Areas of cropland change in China based on four commonly used land use/land cover datasets**

Note: C represents cropland, W represents wood, G represents grassland, RW represents water and residential areas, O represents others. X-Y represents the change from land-use type X to land use type Y.

CNLUCC represents the China Land Use and Land Cover Change Database (https://www.resdc.cn/DOI/DOI.aspx?DOIID=54), CLCD represents the China land cover dataset (https://doi.org/10.5281/zenodo.4417810), ESA represents the European Space Agency-land cover (https://cds.climate.copernicus.eu/), MODIS represents the MCD12Q1 data (https://modis.gsfc.nasa.gov/).

### **References**

1. Liang, D., *et al.* China’s greenhouse gas emissions for cropping systems from 1978–2016. *Sci. Data* **8**, 171 (2021).
